# Multifunctional Copper-Containing Mesoporous Glass Nanoparticles as Antibacterial and Proangiogenic Agents for Chronic Wounds

**DOI:** 10.3389/fbioe.2020.00246

**Published:** 2020-03-31

**Authors:** Thomas E. Paterson, Alessandra Bari, Anthony J. Bullock, Robert Turner, Giorgia Montalbano, Sonia Fiorilli, Chiara Vitale-Brovarone, Sheila MacNeil, Joanna Shepherd

**Affiliations:** ^1^School of Clinical Dentistry, University of Sheffield, Sheffield, United Kingdom; ^2^Department of Applied Science and Technology, Politecnico di Torino, Turin, Italy; ^3^Department of Materials Science and Engineering, University of Sheffield, Sheffield, United Kingdom

**Keywords:** biomaterials, antibacterial, proangiogenic, multifunctionality, glass nanoparticles, mesoporous, chronic wound and burn healing

## Abstract

The physiological wound healing process involves a cascade of events which could be affected by several factors resulting in chronic, non-healing wounds. The latter represent a great burden especially when bacterial biofilms are formed. The rise in antibiotic resistance amongst infectious microorganisms leads to the need of novel approaches to treat this clinical issue. In this context, the use of advanced biomaterials, which can enhance the physiological expression and secretion of the growth factors involved in the wound healing process, is gaining increasing attention as a robust and appealing alternative approach. Among them, mesoporous glasses are of particular interest due to their excellent textural properties and to the possibility of incorporating and releasing specific therapeutic species, such as metallic ions. One of the most attractive therapeutic ions is copper thanks to its proangiogenic and antibacterial effects. In this contribution, copper containing mesoporous glass nanoparticles were proposed as a multifunctional device to treat chronic wounds. The developed nanoparticles evidenced a very high specific surface area (740 m^2^/g), uniform pores of 4 nm and an almost total release of the therapeutic ion within 72 h of soaking. The produced nanoparticles were biocompatible and, when tested against Gram positive and Gram negative bacterial species, demonstrated antibacterial activity against both planktonic and biofilm bacteria in 2D cell monolayers, and in a 3D human model of infected skin. Their proangiogenic effect was tested with both the aortic ring and the chick chorioallantoic membrane assays and an increase in endothelial cell outgrowth at a concentration range between 30 and 300 ng/mL was shown. Overall, in this study biocompatible, multifunctional Cu-containing mesoporous glass nanoparticles were successfully produced and demonstrated to exert both antibacterial and proangiogenic effects.

## Introduction

The rise in antibiotic resistance (AMR) amongst infectious microorganisms is a current and increasing global threat (Shallcross et al., 2015; O'Neill, 2016). There is a dire need for novel or repurposed antimicrobials that do not rely on traditional antibiotics and that will not contribute to AMR.

Metal ions, such as silver and copper, are one such set of antimicrobials that are currently of interest as they can be combined with functional materials for designing healthcare devices (Gorzelanny et al., 2016; Liu et al., 2016; Ramasamy and Lee, 2016; Sun et al., 2016; Bayramov and Neff, 2017). Whilst there is some evidence that bacteria can develop resistance mechanisms to certain forms of copper and silver (Percival et al., 2005; Santo et al., 2011; Finley et al., 2015; Hobman and Crossman, 2015; Williams et al., 2016), they are still promising as antimicrobials due to their broad ranges of biocidal activity against multiple microorganisms, including both Gram positive and Gram negative bacteria. Evidence also exists that metal ions such as silver and copper are either directly antifungal or enhance the actions of antifungal agents (Gomes da Silva Dantas et al., 2018; Radhakrishnan et al., 2018).

One of the main problems with chronic infections is the formation of bacterial biofilms. These are complex, heterogeneous 3D multilayered communities of bacteria encased within a polysaccharide matrix. Traditional antimicrobials can be orders of magnitude less effective against biofilm bacteria than against their “free floating” planktonic counterparts (Koo et al., 2017) for several reasons. The polysaccharide matrix forms a protective barrier which some antibiotics cannot penetrate (Limoli et al., 2015; Flemming et al., 2016), and the biofilm community contains dormant “persister” cells that are not actively dividing and therefore several antibiotic targets, such as cell wall synthesis, are not available (Amato et al., 2014; Brauner et al., 2016). Biofilms can form on almost any surface, organic or inorganic, and biofilms that form within wounds, such as diabetic foot ulcers and burn wounds generate a huge socioeconomic burden (Sen et al., 2009). Therefore, advances in prevention and novel effective approaches for the treatment of such biofilms are important. Toward the development of novel antimicrobial approaches the authors previously established a 3D tissue engineered model of bacterial infected human skin, constructed from a scaffold of decellularised human dermis seeded with human keratinocytes and dermal fibroblasts (MacNeil et al., 2011) which is then thermally injured and infected with bacteria (Shepherd et al., 2009). This model has proven useful in the testing of several novel antimicrobials (Bertal et al., 2009; Shepherd et al., 2011; Zheng et al., 2019) and in performing basic infection studies (Bullock et al., 2019).

Among the wide class of advanced biomaterials, mesoporous bioactive glasses (MBGs) have attracted increasing attention to develop alternative and promising antibacterial strategies, primarily because of their capability to release therapeutic dosages of antimicrobial agents (Kargozar et al., 2018), thanks to the excellent exposed surface area and accessible uniform nanochannels (Vallet-Regí, 2010). More recently, several studies proved that the incorporation of selected therapeutic ions into the MBG framework imparts specific biological functions, such as antibacterial, proosteogenic and proangiogenic effects (Hoppe et al., 2011; Wu and Chang, 2014).

Among the therapeutic ions which can exert multifunctional actions, copper has been extensively investigated with the aim of improving the biological effect of MBGs, due to the recognized proangiogenic effect of copper alongside its antibacterial potential (Wu et al., 2013; Wang et al., 2016). Several studies have proven the antibacterial potential of Cu-substituted MBGs against both planktonic bacteria and biofilms. Wu et al. (2013) tested the antibacterial potential of Cu-containing MBG scaffolds and showed that the number of viable *Escherichia coli* bacteria were significantly reduced after 7 days of incubation, ascribing this effect to the redox potential of copper and the concomitant production of hydroxyl radicals which are detrimental to the bacteria. Consistent with this, the authors recently reported (Bari et al., 2017) that Cu-containing MBG nanoparticles and their ionic extracts exerted promising antibacterial activity against both Gram positive and Gram negative bacteria (*Staphylococcus aureus, Staphylococcus epidermidis* and *E. coli)* and were effective in inhibiting and preventing biofilm produced by *S. epidermidis*.

Since the process of wound healing in soft tissue requires revascularization of the affected area, the stimulation of angiogenesis would be an advantage in a therapeutic setting. Can such ion-substituted MBGs be really multifunctional and simultaneously act as proangiogenic and antimicrobial agents, thus providing novel, effective alternatives to traditional therapeutic approaches?

There are various approaches to study proangiogenic properties of bioactive substances, both *ex-* and *in vivo*. *Ex-vivo*, an aortic ring assay can be used in which aortas (embryonic chicken, mouse, or rat) are excised, cut into rings, embedded into an extracellular matrix gel and treated. The numbers of neo-vessels sprouting from the aortic ring can be quantified (Nicosia and Ottinetti, 1990). Concerning the *in vivo* approaches, there are several other methods available including the extensively used chick chorioallantoic membrane (CAM) assay, widely used since its first development due to the visibility and accessibility of the highly vascularised membrane in fertilized chicken eggs (Nowak-Sliwinska et al., 2014). Using other methods, Lin and co-workers (Lin et al., 2016) demonstrated that the growth of new vessels in the fibrous tissue of rat calvaria defects was promoted by copper ions released from a copper-containing bioactive glass scaffold. Romero-Sánchez et al. (2018) explored the angiogenic effect exerted by the ionic products of both MBG and Cu-containing MBG. The proangiogenic effect of Cu-containing MBGs was confirmed by the increase in vessel number and thickness of the sub intestinal venous plexus (SIVP) in the *in vivo* zebrafish embryo assay.

Moreover, the close connection between osteogenesis and angiogenesis was confirmed by Li and co-workers (Li et al., 2017) through *in vivo* experiments in a goat model after anterior cruciate ligament (ACL) reconstruction which demonstrated that the Cu-substituted bioactive glass could promote bone regeneration.

Following on from this, in this contribution copper-containing MBG nanoparticles are proposed as a multifunctional agent to treat chronic wounds, able to target both antibacterial infections and to promote neovascularisation. To this aim, the authors assessed the antibacterial potential, both in biofilm models and in the infected tissue engineered skin model, and investigated the proangiogenic activity using aortic rings and *in vivo* CAM assays.

## Materials and Methods

### MBG and Cu-Substituted MBG Nanoparticles

Cu-substituted MBGs with the following nominal molar composition 85SiO_2_/13CaO/2CuO %mol were produced by following a synthesis procedure optimized by the authors (Bari et al., 2017). Briefly, 6.6 g of cetyltrimethylammonium bromide (CTAB, ≥ 98%, Sigma Aldrich) and 12 mL of ammonium hydroxide solution (NH_4_OH, Sigma Aldrich) were dissolved by stirring for 30 min in 600 mL of double distilled water. Into this solution, 30 mL of tetraethyl orthosilicate (TEOS, 99%, Sigma Aldrich), 4.888 g of calcium nitrate tetrahydrate (Ca(NO_3_)_2_·4H_2_O, 99%, Sigma Aldrich) and 0.428 of copper chloride (CuCl_2_, 99%, Sigma Aldrich) were dissolved, under stirring for 3 h. Then, the powders were separated by centrifugation at 10,000 rpm for 5 min (Hermle Labortechnik Z326), washed one time with distilled water and two times with ethanol. The final precipitate was dried at 70°C for 12 h. Finally, the powders were calcined at 600°C in air for five at a heating rate of 1°C/min using a Carbolite 1300 CWF 15/5. The obtained calcined powders will be referred hereafter as Cu-MBG.

The same route was followed in order to produce a reference sample without copper with the following nominal molar composition 85SiO_2_/15CaO (%mol) and named hereafter as MBG.

### Physico-Chemical Characterization of MBG and Cu-MBG

The morphology of produced nanoparticles was investigated by Field Emission Scanning Electron Microscopy (FE-SEM) using a ZEISS MERLIN instrument. In order to perform the FE-SEM analysis, 10 mg of powders were dispersed in 10 mL of isopropanol using the Digitec DT 103H (Bandelin) ultrasonic bath for 5 min. Then, 5 μL of the resulting stable suspension were dropped on a carbon coated copper grid and coated with 5 nm of Cr layer. Compositional characterization of the sample was carried out by Energy Dispersive Spectroscopy (EDS; Aztec EDS, Oxford instruments). Wide-angle X-ray diffraction (WA-XRD) investigation of the samples was performed by using the XRD, X'Pert PRO (PANanalytical).

The nitrogen adsorption-desorption measurements at −196°C were performed using an ASAP2020, Micromeritics. Samples were outgassed at 150°C for 5 h before analysis. The Brunauer-Emmett-Teller (BET) equation was adopted to calculate the specific surface area (SSA) from the adsorption data, in the range of 0.04–0.2 p/p_0_. The pore size distribution was calculated by the DFT method (Density Functional Theory) using the NLDFT kernel of equilibrium isotherms (desorption branch).

### Copper Ion Release Test From Cu-MBG

To evaluate the Cu ion release, 5 mg of Cu-MBG were suspended in 20 mL of Tris HCl buffer (Tris(hydroxymethyl) aminomethane (Trizma) (Sigma Aldrich) 0.1 M, pH 7.4) at a final concentration of 250 μg/mL, according to the protocol reported by Shi et al. (2016). The samples were kept immersed for different time points up to 14 days at 37°C in an orbital shaker Excella E24 with an agitation rate of 150 rpm. At each predefined time point (3 h, 24 h, 3 d, 7 d, and 14 d) the suspensions were centrifuged for 5 min at 10,000 rpm (Hermle Labortechnik Z326), half of the supernatant was collected and replaced by the same amount of fresh buffer.

The ion concentration in the supernatant solution was obtained by ICP (ICP-MS, Thermoscientific, ICAPQ), after appropriate dilutions. Each experiment was carried out in triplicate.

### Evaluation of Cytotoxicity and Anti-bacterial Properties

#### Construction of 3D Tissue Engineered Skin

3D tissue engineered skin constructs were prepared as described previously (MacNeil et al., 2011). Briefly, human dermal fibroblasts were isolated from split-thickness skin biopsies received from abdominoplasties and breast reductions performed at the Northern General Hospital, Sheffield in accordance with NHS Research ethics approvals (Ref: 15/YH/0177). Research Ethics approval was obtained from the Sheffield Research Ethics Committee. Donors gave informed and written consent. Cells were maintained in DMEM high glucose (4,500 mg/L glucose), 10% v/v fetal calf serum (FCS), 2 mM l-glutamine, 0.625 mg/mL amphotericin B, 100 IU/ mL penicillin, and 100 mg/mL streptomycin (all Sigma Aldrich, UK). Keratinocytes (HaCaT) cells were purchased from Promega and maintained in Greens media (DMEM high glucose (4,500 mg/L glucose) and Ham's F12 medium in a 3:1 ratio, 10% v/v FCS, 10 ng/mL human recombinant epidermal growth factor, 0.4 mg/mL hydrocortisone, 10–10 M cholera toxin, 18 mM adenine, 5 mg/mL insulin, 5 mg/mL apo-transferrin, 20 mM 3,3,5-tri-idothyronine, 2 mM glutamine, 0.625 mg/mL amphotericin B, 100 IU/mL penicillin, and 1,000 mg/mL streptomycin). Decellularised dermis (DED), produced by removing cells using 1 M NaCl as detailed in MacNeil et al. (2011) from human cadaver skin (Euro Skin, ethical approval for research not required by user), was used as a base scaffold. Rings of DED (15 mm in diameter) were cut and placed within 12 mm tissue culture inserts with 4 mm pores in the base (Greiner). Inserts were suspended from the edges of 12 well plates into the wells. Greens medium containing 10% fetal calf serum was added to the bottom of the wells so that it lapped the under surface of the DED. The DED was then seeded with 1 × 10^5^ fibroblasts and 5 × 10^5^ keratinocytes, each in 250 mL of 10% Greens medium. Again, after 24 h incubation at 37°C, seeding medium was removed and replaced with fresh Greens. After a further 24 h, medium was removed from inside the inserts in order to leave the constructs at an air/liquid interface. Greens medium in the tissue culture wells was replaced every 24 h and constructs were used for experimentation after 14 days at air/liquid interface.

#### Cytotoxicity Testing

The toxicity of two concentrations of MBG and Cu-MBG was tested using monolayers of human primary fibroblasts isolated from excess skin from abdominoplasties as described previously (MacNeil et al., 2011). Human primary fibroblasts were seeded at a density of 100,000 cells per well in a 24 well sterile tissue culture plate in 1 mL of Dulbecco's minimal essential medium (DMEM) (Sigma Aldrich) and incubated overnight at 37°C/5% CO_2_ to form a monolayer. MBG and Cu-MBG were added to the wells with fresh media to a final concentration of either 0.1 or 1 mg/mL in 1 mL of total suspension. Dimethyl sulphoxide (DMSO, Sigma Aldrich) was used as a positive toxicity control with 0.5 mL added into the well. Cells without any treatment were used as negative toxicity controls. Plates were then incubated for 48 h at 37°C/5% CO_2_ and then analyzed using the metabolic assay PrestoBlue™. For the assay, all medium was removed and 700 μL of a 1:10 dilution of PrestoBlue:DMEM were added for 1 h. 300 μL in duplicate were removed from the 24 well plate and added to a 96 well plate for reading. Fluorescence was then read with excitation/emission wavelengths of 560 nm/590 nm using a Tecan spectrophotometer.

3D skin samples were incubated in 1 mL of Greens media with 0.1 mg/mL Cu-MBG suspensions. As reference material, Acticoat Flex 3 (Smith & Nephew), a commercial coating for infected wounds, was cut in 5 × 5 mm sections and incubated in the same conditions. Samples were incubated for 24 h at 37°C/5% CO_2_. After the removal of the incubation medium and of Acticoat Flex 3 sections, 3D skin samples were washed with phosphate buffered saline (PBS) and tested for cell metabolism using the PrestoBlue assay, as above.

#### Bacteria

Antibacterial properties of nanoparticles were tested against clinical isolates of *S. aureus* (S235) and *Pseudomonas aeruginosa* (SOM-1), representative of Gram positive and Gram negative bacteria respectively that commonly infect chronic skin wounds. Both species were cultured in brain heart infusion (BHI) broth (Oxoid, UK) at 37°C overnight and stored at 4°C on BHI agar (Sigma Aldrich, UK).

#### Infection of Skin Constructs

Constructs were washed in antibiotic-free Greens medium for 72 h prior to infection. They were then burnt using application of a surgical cauterizer for 8 s immediately prior to infection in order to provide the bacteria with a mode of entry into the dermal tissue. 1 × 10^6^ *P. aeruginosa* or *S. aureus* cells in 20 μL BHI broth per construct were pipetted into the inserts covering the epidermal surface. Infected constructs and non-infected controls were incubated at 37°C/ 5% CO_2_.

#### Measurement of Antibacterial Activity Against Planktonic Bacteria

The antibacterial activity of both Cu-MBG and MBG nanoparticles against planktonic *S. aureus* S235 and *P. aeruginosa* SOM-1 was determined by suspending the particles at a concentration of 100 μg/mL in sterile phosphate buffered saline (PBS) and incubating the resulting suspension with bacterial broth cultures. 100 μL of nanoparticle suspension at 100 μg/mL concentration were incubated at 37°C with 100 μL of a suspension of bacteria at a concentration of ~1 × 10^10^ colony forming units per mL (cfu/mL). Following incubation for 24 h, serial dilutions of each sample were performed, and viable counts carried out according to the standard Miles & Misra technique by spotting out dilutions onto BHI agar plates and counting the numbers of cfu/mL after incubating plates for 24 h at 37°C.

#### Measurement of Antibacterial Activity Against Bacterial Biofilms

To test antibacterial activity against bacterial biofilms, two techniques were used to measure both prevention of biofilm formation and biofilm disruption. Clinical isolates of *P. aeruginosa* and *S. aureus*, as detailed above, were used to form 48 h biofilms in BHI broth on tissue culture plastic. Overnight cultures of bacteria were diluted 1:100 in BHI broth and incubated at 37°C for 48 h, either with addition of 0.1 mg/mL Cu-MBG at the time of inoculation (to test prevention of biofilm formation) or after 48 h when the biofilms had formed (to test biofilm disruption). In the case of biofilm disruption, 48 h biofilms were incubated with 0.1 mg/mL Cu-MBG suspensions for 24 h before performing assays as follows:

- Absorbance of the whole sample (planktonic and biofilm bacteria) was read at 600 nm to measure the entire biomass using a Tecan spectrophotometer with Magellan software. Following this, planktonic bacteria were removed and the absorbance at 600 nm ensured the measure of the biofilm mass alone;
- To measure metabolic activity, PrestoBlue assays were run on the biofilm according to manufacturer's instructions. Briefly, biofilms cultured in 12 well plates were incubated with 1 mL 1xPrestoBlue reagent (ThermoFisher) at 37°C for 1 h. Fluorescence was then measured with excitation/emission wavelengths of 560 nm/590 nm using a Tecan spectrophotometer as above.

#### Antibacterial Activity in Infected 3D Skin Models

The disruption of biofilm forming ability was tested by adding 0.1 mg/mL of Cu-MBG suspension to the skin simultaneously to the bacteria and incubated for 24 h before measuring bacterial viability. To investigate if the samples could disrupt a pre-existing biofilm the samples were added to the skin 24 h after infection with bacteria. The skin was then left incubating at 37°C/5%CO_2_ for a further 24 h before surviving bacterial numbers were counted. At the testing point the tissue was weighed, then homogenized in 1 mL BHI broth. The resulting homogenates were diluted serially and used to perform viable counts of bacteria in the samples using standard Miles & Misra methods as above.

### Proangiogenesis Effect

#### Aortic Ring Assay

Aortas from 12-days old chicken embryos were harvested under sterile conditions and cut into 4–5 rings of <1 mm length after removing fat and membranous tissue from around the vessel (~4–5 mm long). Rings were embedded in 80 μL of extracellular matrix gel (ECM gel, Sigma Aldrich) in wells of 48 well plates. After allowing the ECM gel to gel at 37°C for 20 min, alpha MEM media (Sigma Aldrich) supplemented with 2% FCS (Sigma Aldrich) was gently added to the well.

The dissolution extracts of MBG and Cu-MBG were prepared by suspending the powder at different concentrations (1 mg/mL, 300 μg/mL, 100 μg/mL, and 30 μg/mL) in alpha MEM growth media at 4°C for 72 h. After the incubation the powders were removed from the suspension by filtration, and the resulting solution extracts were used for the aortic ring experiments. VEGF was used as a positive control to treat aortic rings at 3, 10, and 30 ng/mL dosage.

Outgrowth from rings was captured by photography (40x magnification) every 24 h for 5 days and 48 h was chosen as a time point to compare outgrowth between the tested particle extracts and controls. Outgrowth was measured by tracing the outline of the area covered by the outgrowth of endothelial sprouts/network then subtracting the area covered by the original aortic ring using ImageJ software.

#### CAM Assay

Chicken eggs obtained within 24 h of laying were incubated for 72 h at 37°C, then removed from their shells into sterile containers placed inside Petri dishes. After verifying the presence of a living embryo, the *ex ovo* eggs/embryo were incubated for a further 4 days at 37°C. Both MBG and Cu-MBG were applied to the CAM membrane. 100 μL of particle suspension bearing 1 mg of nanomatrix powder in PBS were placed within a 0.7 mm diameter polypropylene ring on the CAM membrane, and the egg incubated for a further 3 days. The CAM membrane around the ring bearing the nanoparticle treatment was then photographed. To quantify the change in vasculature, green channel images of the CAM were created from the original RGB (red, green, blue) images. These were then analyzed using Angiotool (National Cancer Institute, Washington USA). Data is presented as an average of three experiments, each using six eggs per treatment group, ± Standard Error of the Mean (SEM). Statistical significance was determined by *T*-test, and *p* < 0.05 deemed to be significant.

## Results

### Nanoparticle Characterization

**Figure 1:**
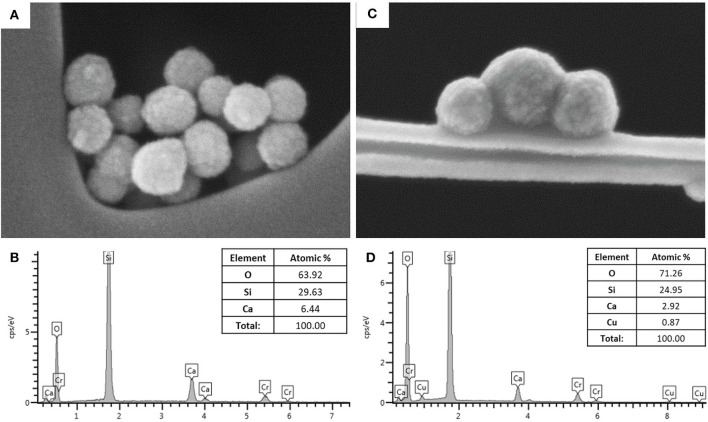
**(A)** FE-SEM image of MBG, **(B)** EDS spectrum of MBG; **(C)** FE-SEM image of Cu-MBG, **(D)** EDS spectrum of Cu-MBG.

MBG and Cu-MBG morphology was explored by FE-SEM which revealed uniform spherical particles, with sizes ranging between 100 and 150 nm, as shown in Figures 1A,C. The EDS spectra of the powder dispersed on carbon tape (Figures 1B,D) confirmed the theoretical composition of the samples. In particular, the EDS investigation of Cu-MBG (Figure 1D) suggested that the incorporation of copper inside the framework successfully occurred with a Cu/Si molar ratio (as an average of three measurements) very close to the nominal ratio.

The amorphous state of both MBG and Cu-substituted MBG was confirmed by WA-XRD analysis (data not shown) which evidenced only the broad peak at around 20° 2θ, typical of amorphous silica-based systems.

**Figure 2:**
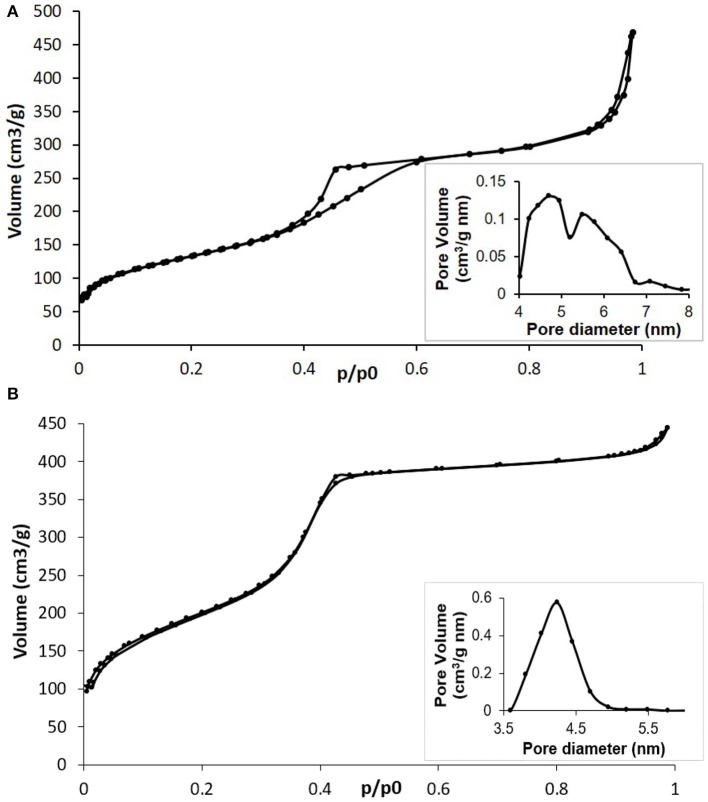
**(A)** N_2_ adsorption-desorption isotherm of MBG and related DFT pore size distribution (inset); **(B)** N_2_ adsorption-desorption isotherm of Cu-MBG and related DFT pore size distribution (inset).

The N_2_ adsorption-desorption measurements confirmed the mesoporous structure of both MBG (Figure 2A) and Cu-MBG (Figure 2B) which show a IV type isotherm. Cu-MBG (inset in Figure 2B) displays a single-mode pore size distribution centered at 4 nm, whereas the hysteresis loop in Figure 2A indicated a broad pore size distribution for MBG, confirmed also by the inset in Figure 2A.

The SSA and pore volume of the obtained systems are listed in Table 1. The very high values, especially for Cu-MBG, demonstrated that the incorporation of a metal ion did not negatively affect the formation of the mesoporous structure, as with data reported for similar systems (Bari et al., 2017; Fiorilli et al., 2018). This is in good accordance with data reported by Wang et al. (2016) who demonstrated that the incorporation of copper up to 2% mol concentration improved the structural features of MBGs by inducing an increase of the specific surface area and pore volume.

**Table 1:** Structural features of Cu-MBG.

**Name**	**Specific surface area (m^**2**^/g)**	**Pore size (nm)**	**Volume (cm^**3**^/g)**
MBG	476	4.5–5.5	0.57
Cu-MBG	740	4	0.65

The capability of Cu-MBG to release the therapeutic ions was confirmed by soaking the powder in Tris HCl. In agreement to that reported by one of the authors group (Pontremoli et al., 2018), a burst release was encountered within the first 3 h of incubation and almost the total amount of incorporated copper was released after 3 days of soaking, reaching a concentration of 4.5 ppm.

### Cytotoxicity

**Figure 3:**
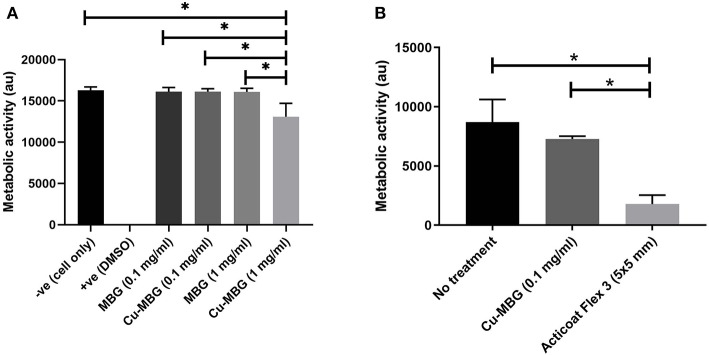
Toxicity of Cu-MBG tested against mammalian cells. **(A)** Toxicity of two different concentrations of MBG and Cu-MBG against a monolayer of human primary fibroblasts over 48 h exposure assessed by metabolic activity measured using a PrestoBlue assay. **(B)** Toxicity of Cu-MBG and of industrial standard silver dressing Acticoat Flex 3 on tissue engineered skin models after 24 h of exposure in model assessed by metabolic activity measured using a PrestoBlue assay. Mean ± standard deviation, ANOVA test used with *p < 0.05.

At 1 mg/mL, MBG nanoparticles were not toxic to human cells tested, but Cu-MBG nanoparticles at 1 mg/mL reduced cellular metabolic activity by ~20% (Figure 3A). No significant difference in metabolism to MBG treated cells was found for Cu-MBG at a concentration of 0.1 mg/mL. This defined the concentration used for the remainder of the study and thus a 0.1 mg/mL concentration was then tested on a more complex model, the tissue engineered skin. The toxicity of Cu-MBG at 0.1 mg/ml concentration was well-tolerated, whereas in comparison a commercially available silver-containing dressing (Acticoat Flex 3) markedly inhibited cell viability in the 3D tissue engineered skin model (Figure 3B).

### Antibacterial Effects Against Planktonic Bacteria

**Figure 4:**
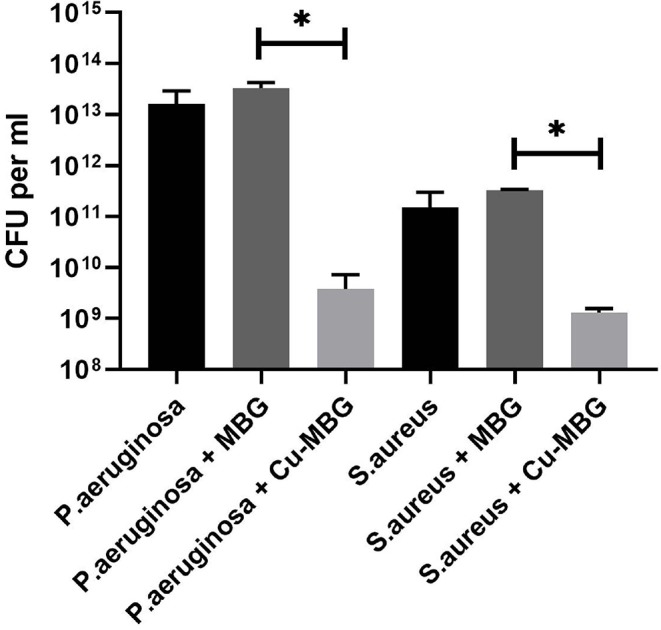
Antibacterial effects of Cu-MBG against planktonic bacteria. Colony forming units per mL of broth were measured following 24 h incubation of bacteria in suspension +/– 100 μg/mL Cu-MBG or MBG. There was a decrease in viable numbers of both species with Cu-MBG—a 3.5-log reduction in viable *P. aeruginosa* and 1.2-log reduction in *S. aureus*. No reduction in bacterial numbers was measured after incubation with unloaded MBG particles. *n* = 3, error bars = SEM, *t*-test performed. *p < 0.05.

Cu-MBG at 100 μg/mL had significant antibacterial effects against clinical isolates of both *P. aeruginosa* and *S. aureus* when in planktonic form, with 1.2–3.5 log reductions in viable numbers of bacteria from control (PBS treated) samples (Figure 4). No antibacterial effect was seen with unloaded MBG nanoparticles, so these were not tested in further antibacterial assays.

### Antibacterial Effects Against Biofilms

**Figure 5:**
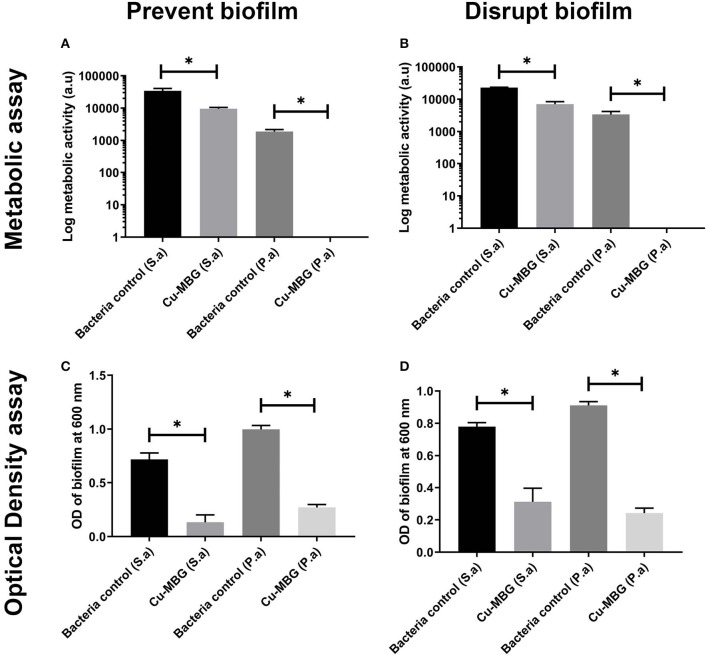
Antibiofilm testing using Cu-MBG samples, measured using biofilm bacteria viability (metabolic assay) **(A,B)** and biofilm biomass (optical density) **(C,D)**. **(A)** Cu-MBG preventative action on the formation of biofilms using the measure of bacterial metabolic activity. **(B)** Cu-MBG ability to disrupt pre-formed biofilms using the measure of bacterial metabolic activity. **(C)** Cu-MBG preventative action on the formation biofilms using the measure of optical density of the biofilm with higher values indicating greater biofilm formation. **(D)** Cu-MBG ability to disrupt a pre-formed biofilm using the measure of optical density of the biofilm with higher values indicating greater biofilm formation. *n* = 3, means ± standard deviation, ANOVA statistics used with *p < 0.05.

For both species of bacteria tested, exposure to Cu-MBG reduced the existing biofilm (Figures 5A,C) or helped prevent its formation (Figures 5B,D) when considering both biofilm biomass and biofilm metabolic activity. Metabolic activity was greatly reduced for *P. aeruginosa* and to a lesser, but still significant extent, for *S. aureus* after treatment with Cu-MBG. Optical density measurements (Figures 5C,D) revealed that the mass of biofilm present after treatment is reduced for both species. The remaining *P. aeruginosa* biofilms after treatment appear to contain no metabolically active cells after treatment.

### Antibacterial Action of Cu-MBG in a Model of Infected Skin

**Figure 6:**
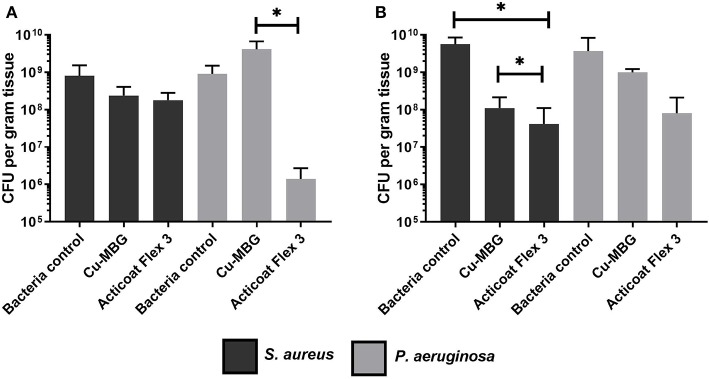
Tissue engineered infection skin model testing Cu-MBG activity against the formation and disruption of biofilms of two different bacterial species. **(A)** Investigation of the Cu-MBG ability to prevent a biofilm formation on the skin models when tested with *S. aureus* and *P. aeruginosa*. **(B)** Investigation of the Cu-MBG ability to disrupt a pre-existing biofilm growing on a tissue engineered skin model when tested against *S. aureus* and *P. aeruginosa*. *n* = 3, plotting mean ± standard deviation, ANOVA statistics used. *p < 0.05.

In contrast to the microbiology results on biofilm reported above, the impact of Cu-MBG against *P. aeruginosa* was far less pronounced than its action against *S. aureus* in the infected skin model. No statistical significance was found between *P. aeruginosa* controls and the treated samples (Figure 6). While the industrial standard Acticoat Flex 3 was more effective than Cu-MBG against *P. aeruginosa* infection in a 3D skin model, it was of similar effectiveness against *S. aureus* for both prevention and disruption of biofilms in the infected skin model.

### Assessment of Proangiogenic Activity of Cu-MBG

#### Aortic Ring Assay

**Figure 7:**
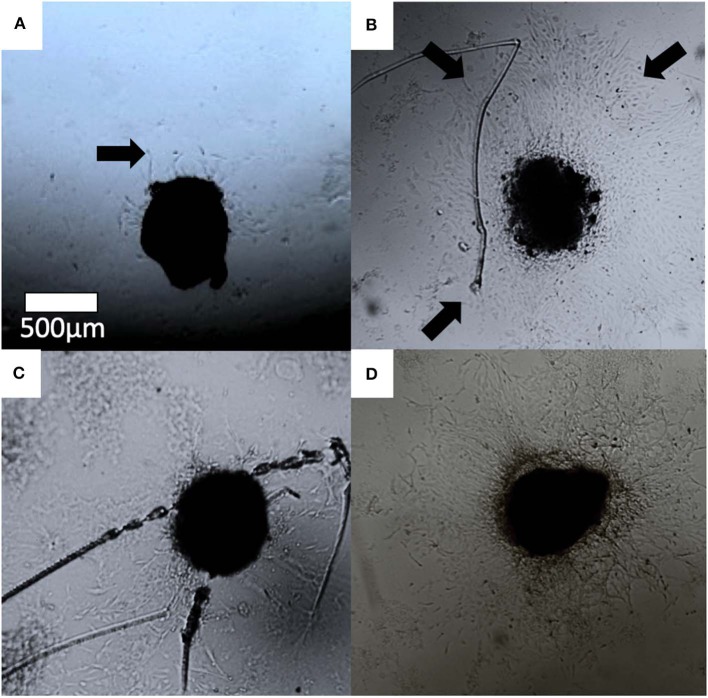
Optical images of aortic ring cell outgrowth in **(A)** control, **(B)** 30 ng/ml VEGF **(C)** MBG, and **(D)** Cu-MBG treated aortic rings. Arrows indicate examples of outgrowth.

Endothelial cell outgrowth began by 24 h for the majority of aortic rings but was prominent enough to be measured by 48 h. Cell outgrowth from the aortic rings formed branching networks (Figure 7). By 72 h, many wells had outgrowths which extended beyond the field of view, making accurate measurement difficult. For this reason, we chose a set time point of 48 h for initial calculations. The data is presented as the area of endothelial outgrowth in mm^2^.

**Figure 8:**
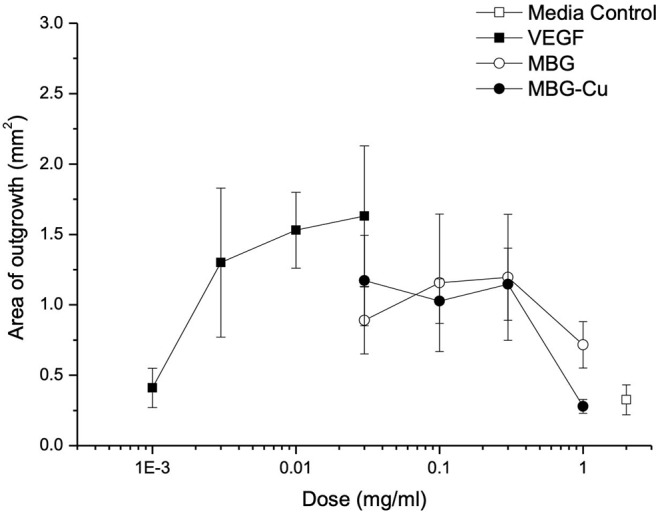
Response of aortic rings to treatment with VEGF, MBG and Cu-MBG. Data is an average of three (MBG) and six (Cu-MBG) experiments ±/– SEM.

VEGF was used as a positive control in the aortic ring assay. Treatment promoted an increase in the rate of cell outgrowth, which appeared to be maximal by 10 ng/mL VEGF (Figure 8). The area of outgrowth increased from 0.3 to 1.5 mm^2^ with the addition of VEGF representing an ~5-fold increase in the area of outgrowth. With both MBG and Cu-MBG there was an increase in outgrowth with doses of 30 to 300 μg/mL, compared to control media. A decrease in outgrowth was observed at 1 mg/mL for both MBG and Cu-MBG (Figure 8).

#### Assessment Using the CAM Assay

**Figure 9:**
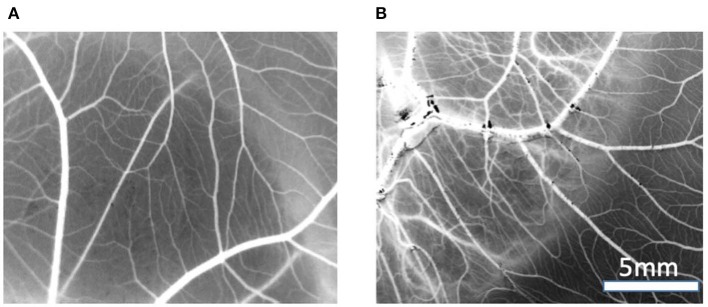
Example of vasculature visible on CAM, images are processed to black and white for data processing, treated with **(A)** control MBG, **(B)** Cu-MBG.

Image analysis of the CAM membranes yielded data on the effect of MBG and Cu-MBG on a number of parameters, specifically the total area occupied by vessels, the total length of the imaged vessels, the average length between branching points, and the number of branching points. Vessels over 20 μm in diameter are described as large vessels, those under 20 μm in diameter are described as small vessels. Controls were the CAM with no treatment compared to CAM treated with 1 mg MBG or Cu-MBG. Example images are shown in Figure 9.

**Figure 10:**
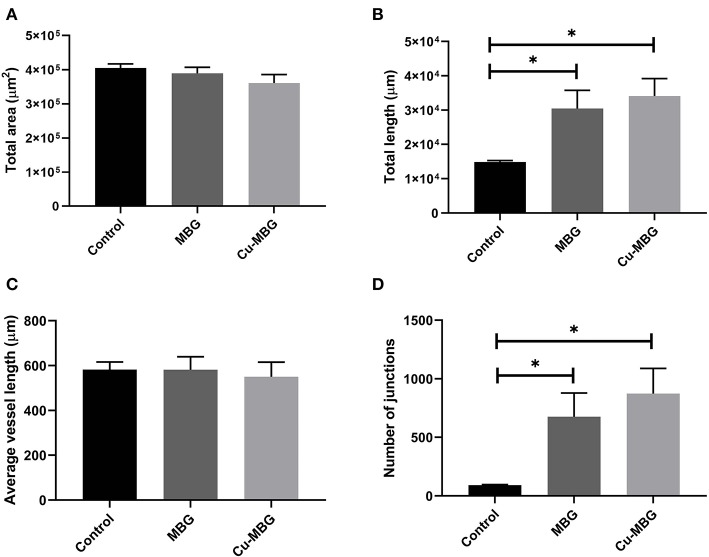
Effect of MBG and Cu-MBG nanoparticles on large vessels (over 20 μm in diameter). **(A)** Total area of vessels per image, **(B)** Total length of vessel per image, **(C)** Average length of vessels between junctions, and **(D)** Number of junctions per image. Data is an average of three experiments, each using six eggs per treatment group, ± SEM. Statistical significance determined by *T*-test, *p < 0.05.

**Figure 11:**
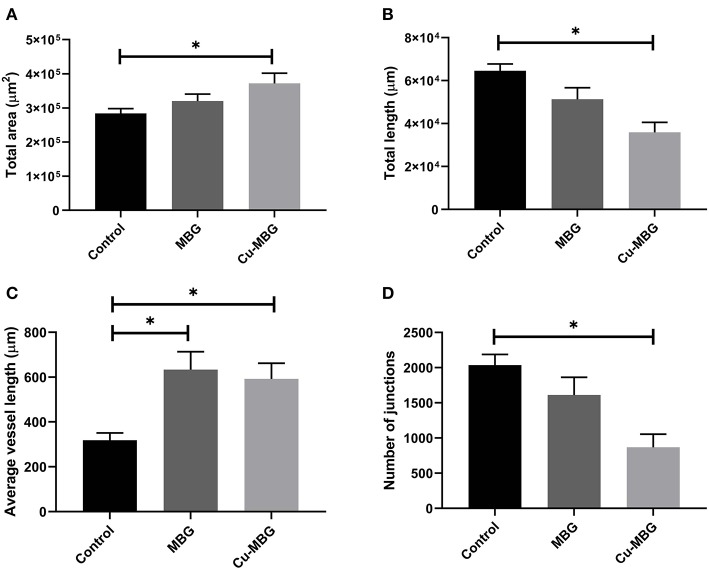
Effect of MBG and Cu-MBG nanoparticles on small vessels (under 20 μm in diameter). **(A)** Total area of vessels per image, **(B)** Total length of vessel per image, **(C)** Average length of vessels between junctions, and **(D)** Number of junctions per image. Data is an average of three experiments, each using six eggs per treatment group, ± SEM. Statistical significance determined by *T*-test, *p < 0.05.

With respect to large vessels there was, a significant increase in the total vessel length and the number of junctions in both MBG and Cu-MBG treated CAM membranes (Figures 10C,D). There was no significant effect of any treatment on the total area covered by the large vessels or in the average length of the vessels (Figures 10A,B). For small vessels, a different picture emerged. There was a small increase in total vessel area with Cu-MBG which was accompanied by a significant decrease in the total vessel length (Figures 11A,B). There was a significant increase in the average small vessel length for both MBG and Cu-MBG (Figure 11C), and a significant fall in the number of junctions for Cu-MBG but not MBG (Figure 11D).

## Discussion

Our aim was to develop multifunctional nanoparticles to treat chronic wounds. To do this, in this study we tested the ability of copper containing mesoporous glass nanoparticles against bacterial infection and for their ability to promote angiogenesis.

MBG nanoparticles containing 2% mol of copper were successfully obtained through a base-catalyzed sol-gel route. The incorporation of copper did not negatively affect the mesoporous structure and the textural features of the particles, characterized by specific surface area and a pore volume remarkably higher than those of not-templated sol-gel glasses (Bari et al., 2017). The copper releasing capability for up to 14 days was confirmed quantitatively by ICP analysis, which evidenced an almost total release after 72 h of soaking.

The effective antibacterial concentration of copper in the Cu-MBG nanoparticles used was not cytotoxic to skin cells in monolayer or to 3D human tissue engineered skin constructs. With respect to the antimicrobial studies, our data showed that the commercially available silver releasing Acticoat Flex 3 was as effective or sometimes more potent than Cu-MBG in both prevention and disruption of biofilms in the infected skin model. However, it was also more cytotoxic than the Cu-MBG nanoparticles. In clinical use, this degree of cytotoxicity is known to cause problems as there is only a small concentration difference between the concentration of silver required to act as an antimicrobial and the concentration which inhibits wound healing. Given the concentration of Cu^2+^ ions released by the Cu-MBG, there is potential in future to use a slightly increased concentration of these nanoparticles to increase the antimicrobial activity without inducing deleterious cytotoxicity.

Turning next to the desirability of inducing new vasculature we were able to obtain some encouraging results on the effects of Cu-MBG nanoparticles in stimulating angiogenesis. Many chronic wounds suffer from poor vasculature and there have been decades of research trying to develop biomaterials to promote angiogenesis. VEGF is the best known and most potent of the cascade of proangiogenic growth factors. However, topical application of VEGF has proved disappointing clinically, and this protein is labile and expensive. Thus, it is not being pursued clinically, but it is routinely used as a positive control in experimental studies of angiogenesis.

Copper ions have previously been shown to have a proangiogenic effect (Zhao et al., 2015) and in the present work we extended this proangiogenic study in a simple *in vitro* assay of angiogenesis, the chick aortic ring assay—where outgrowth of endothelial cells from rings of embryonic aorta gives a measure of the degree to which the candidate material promotes angiogenesis. We found that there was an overall trend for a proangiogenic effect for both the MBG and Cu-MBG nanoparticles. Both MBG systems showed outgrowth between 50 and 75% of that seen with VEGF at 30 ng/mL. At a dose of 1 mg/mL the fall off in outgrowth was more marked where Cu was present, which could be due to toxicity of Cu to cells. In fact, our cytotoxicity data in this study demonstrated that at 1 mg/mL, Cu-MBG were toxic to primary human fibroblasts in monolayers. The increase in endothelial cell outgrowth even with MBG may be due to the release of silicon ions. Silicate ions have been widely reported to induce angiogenesis (Dashnyam et al., 2017a,b). While both Si and Cu are reported to increase angiogenesis, their content in the sample differs by more than one order of magnitude. The high concentration of Si released from the MBG could mask any proangiogenic effects due to an increase in Cu concentration; hence we see an increased proangiogenic effect from the Cu only when the Cu-MBG are at a low concentration. Nonetheless, the Cu-MBG nanoparticles also exhibit antibacterial properties.

Data from the CAM assay shows an increase in both the total length and number of junctions of large vessels in response to the presence of MBG, which were further increased in the case of Cu-MBG. This proangiogenic effect may be a combination of the effect of Si and Cu ions as discussed. Future studies may include a more detailed analysis of the pro-angiogenic properties of the ions, for example by detection of angiogenic markers expressed in vessels exposed to the ions by flow cytometry. The results in small vessels show effects on the vessel area and length, however it is difficult to interpret these data. Cu released from Cu-MBG lead to an increase the visible area of blood vessels and the average vessel length between junctions. However, there was also a fall in both the total vessel length and the number of junctions –while this may seem counterintuitive, perhaps indicating that the population has an increased average vessel diameter.

Cu-MBG displayed effective antibacterial properties against clinical isolates of both Gram positive and Gram negative bacteria, lending multifunctional aspects to these nanoparticles. MBG without the addition of copper showed no measurable antibacterial activity against either species used here, suggesting that the antibacterial effects arise from the copper alone. In a previous work (Bari et al., 2017) we reported the antibacterial activity of the Cu-MBG against *S. epidermidis* and *E. coli* as well as *S. aureus*, and the current results are consistent with the antimicrobial action against both Gram positive and Gram negative species tested previously published. Our microbiology data suggests that the Cu-containing MBG nanoparticles are more effective against *P. aeruginosa* than *S. aureus*. In this study we expanded the antimicrobial work further by using infected 3D models of tissue engineered human skin. In contrast to results performed in tissue culture plastic, we found that, when using the 3D model, *P. aeruginosa* were more resistant to treatment than *S. aureus*. We postulate that this could be as a result of the ability of *P. aeruginosa* to penetrate more deeply into the deep dermal layers of tissue engineered skin, as previously observed by our group (Shepherd et al., 2009; Zheng et al., 2019) thus escaping the surface treatment. This supports the absolute necessity for the use of more physiologically relevant 3D models in antimicrobial research, rather than relying on 2D cell cultures or bacterial biofilm in isolation of a relevant biological situation.

Other groups have developed multifunctional copper-containing particles; for example Huang et al. (2017) have modified the surface of titanium implants with a chitosan-gelatin nanocomposite containing Cu ions. These composites also have antibacterial and proangiogenic properties, however there are limitations with respect to the usage since they are linked to titanium. The nanoparticles described in this work are not bound to a surface and could have a wide variety of potential applications. For instance, we have recently shown that the MBG nanoparticles can be incorporated into a thermosensitive injectable polyurethane gel allowing targeted delivery of therapeutic ions (Pontremoli et al., 2018).

## Conclusion

In this contribution, multifunctional copper-containing mesoporous glass nanoparticles were obtained through a base-catalyzed sol-gel method. The spherical nanoparticles, characterized by a large surface area and tunable pore size, were able to release copper in a predictable way over 14 days. They showed a remarkable antibacterial activity against both planktonic and biofilm bacteria with a broad spectrum of antimicrobial action, whilst showing no cytotoxicity in either 2D cell monolayers or a 3D human skin model unlike a comparative commercial dressing. A clear proangiogenic effect of both MBGs and Cu-containing MBGs was confirmed by an increase in endothelial cell outgrowth seen at concentrations between 30 and 300 μg/mL.

Overall, this study demonstrated the feasibility of producing biocompatible multifunctional therapeutic Cu-containing MBGs which are able to exert both antibacterial and proangiogenic effects and, their appealing potential in development of multifunctional devices.

## Data Availability Statement

The datasets generated for this study are available on request to the corresponding author.

## Ethics Statement

Research Ethics approval was obtained from the Sheffield Research Ethics Committee. Donors gave informed and written consent.

## Author Contributions

CV-B, SF, and SM conceived the presented work. CV-B, SM, and JS supervised the overall work. TP, AB, AJB, RT, GM, SF, and JS all performed experiments and analyzed data. AB, GM, and SF prepared and tested the nanoparticles. TP, AJB, RT, and JS performed antibacterial and proangiogenic tests on the materials. All authors discussed the results and contributed to the final manuscript.

### Conflict of Interest

The authors declare that the research was conducted in the absence of any commercial or financial relationships that could be construed as a potential conflict of interest.
